# From superhydrophobicity to icephobicity: forces and interaction analysis

**DOI:** 10.1038/srep02194

**Published:** 2013-07-12

**Authors:** Vahid Hejazi, Konstantin Sobolev, Michael Nosonovsky

**Affiliations:** 1 College of Engineering & Applied Science, University of Wisconsin-Milwaukee, Milwaukee, Wisconsin 53211, United States

## Abstract

The term “icephobicity” has emerged in the literature recently. An extensive discussion took place on whether the icephobicity is related to the superhydrophobicity, and the consensus is that there is no direct correlation. Besides the parallel between the icephobicity and superhydrophobicity for water/ice repellency, there are similarities on other levels including the hydrophobic effect/hydrophobic interactions, mechanisms of protein folding and ice crystal formation. In this paper, we report how ice adhesion is different from water using force balance analysis, and why superhydrophobic surfaces are not necessary icephobic. We also present experimental data on anti-icing of various surfaces and suggest a definition of icephobicity, which is broad enough to cover a variety of situations relevant to de-icing including low adhesion strength and delayed ice crystallization and bouncing.

The terms “icephobic” and “icephobicity” have been used in the literature recently^1^,^2^,^3^,^4^,^5^,^6^,^7^,^8^,^9^,^10^,^11^, although the words are relatively new and still absent from the Oxford English Dictionary. The keyword “icephobic” was used for the first time by Kulinich and Farzaneh^3^ as well as in some industrial reports^12^,^13^. Next time, in the form “ice phobic,” it appears in 2008 in the work done at NASA^14^. After that, the term appears many times in the literature. The term is analogous to the hydrophobicity and other “-phobicities” (oleophobicity, lipophobicity, omniphobicity, amphiphobicity, etc); however, an exact thermodynamic definition of icephobicity is missing from the literature. The extensive controversy among scholars on whether the icephobicity is related to the superhydrophobicity came to a conclusion that there is no direct correlation^5^,^6^,^7^. In recent publications there are at least three different approaches to the characterization of surface icephobicity. First, icephobicity implies low adhesion force between ice and a solid surface. In most cases, the critical shear stress is calculated, although the normal stress is used as well. The researchers call “icephobic” surfaces with the shear strength between 150 kPa and 500 kPa^2^,^15^ and even as low as 15.6 kPa^16^. Second, some scholars define icephobicity as the ability to prevent ice formation on the surface. Such ability depends on whether a droplet of supercooled water (below the normal freezing temperature of 0°C) freezes at the interface and it can be characterized by time delay of heterogeneous ice nucleation^5^,^17^,^18^. The mechanisms of droplet freezing are quite complex and depend on the temperature level, on whether cooling is performed from the side of the solid substrate or from vapor, and on other factors. Third, an impact test for bouncing-off droplets was suggested^4^ implying that icephobic surfaces repel incoming small droplets (e.g., of rain or fog) at the temperatures below the freezing point.

These three definitions of icephobicity correspond to three different, although related, properties of anti-icing surfaces: they should (i) prevent freezing of water condensing on the surface (ii) prevent freezing of incoming water (iii) if ice formed, it should have weak adhesion strength with the solid, so that it can be easily removed. Mechanical properties of ice and the substrate also of great importance since ice shedding occurs as fracture, either in the mode I (normal) or mode II (shear) cracking, so that crack concentrators are major contributors to the reduced strength^6^.

All of the above considerations suggest that a universal definition of the icephobicity can be a difficult task and the parallel with the superhydrophobicity should be examined in depth. The superhydrophobicity is defined by the water contact angle (CA) > 150° and by low CA hysteresis^19^,^20^, although very high CA can co-exist with high CA hysteresis (the rose petal effect)^21^. Low CA hysteresis corresponds to shear mode of loading at the solid-water interface while a high CA corresponds to the normal loading. The ability to bounce-off incoming droplets constitutes the third aspect of the superhydrophobicity^22^. In the present paper, we will examine adhesion properties of ice in view of their parallelism with the above-mentioned properties of superhydrophobic materials.

## Results

**Figure 1:**
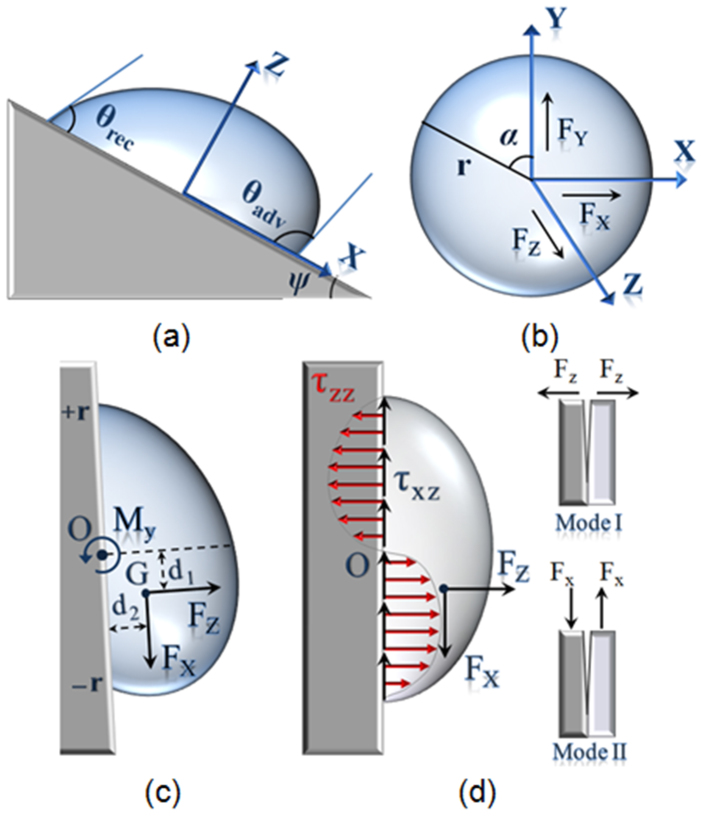
Schematic of the water droplet and ice on the surface. (a) Side view of the water droplet placed on the surface with the tilt angle *ψ*. (b) Top view of a water droplet placed on the surface. (c) Side view of the water droplet placed on the tilted surface and forces and the moment applied to it. (d) Side view of the ice formed on the vertical surface, forces applied to it. Ice detachment occurs as the mode I (crack opening) and mode II (shear) fracture.

First, we studied the parallelism between the dewetting and deicing. These are essentially mechanical processes involving three-phase line “friction” due to CA hysteresis^23^ and mode I or mode II fracture^6^, respectively. To account for these effects we need to write the balance equations for mechanical forces and moments acting on the droplet and on ice particle. For a water droplet placed on a tilted surface (Fig. 1a), we approximate the CA as 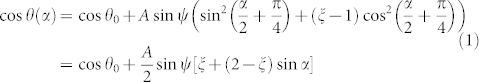
where *θ*_0_ is the water CA on a horizontal surface, *ψ* is the tilt angle, *α* is the angle of the position on the triple line in a cylindrical coordinate system with z-axis as its longitudinal axis (Fig. 1b), *A* is a constant which characterizes the amplitude of CA hysteresis. Note that, in general, the increase of the maximum (advancing) CA is not necessarily equal to the decrease of the minimum (receding) CA. To characterize this asymmetry, we introduce the parameter −1 < ξ < 1. The symmetric case corresponds to ξ = 0 where the change in advancing and receding CAs is equal.

The surface tension of a droplet placed on a solid surface at each point on the triple line can be presented in the vector form as (Supplementary; Surface tension) 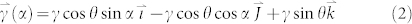
where *γ* is the water-air interfacial energy (about 0.072 Jm^−2^ at room temperature), while the CA hysteresis (the difference between the cosines of advancing and receding CA, Supplementary; CA hysteresis) is 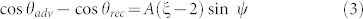
And the force and moment balance equations are (Supplementary; Force and moment balance) 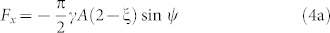

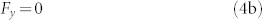

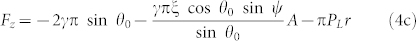

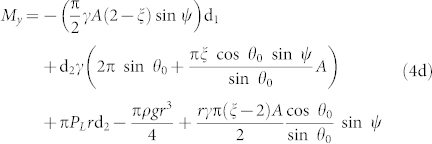
where *P_L_* is the Laplace pressure.

**Figure 2:**
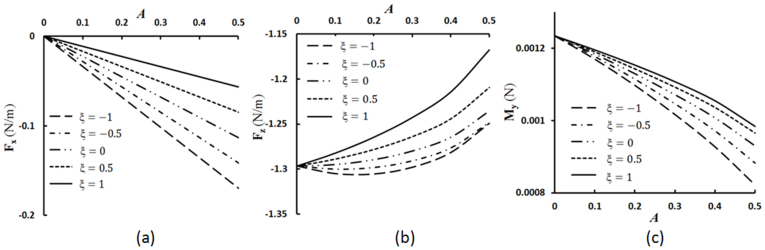
Force and moment versus *A*. (a) *F_x_* versus the constant *A* for various values of ξ (b) *F_z_* versus the constant *A* for various values of ξ (c) The moment versus the constant *A* for various values of ξ and *γ* = 0.072 Jm^−2^ for *θ*_0_ = 60°, *ψ* = 90°, P_L_ = 144 Pa and *r* = 2, *d*_1_ = 1, *d*_2_ = 1 mm.

Figure 2 shows *F_x_*, *F_z_* and *M_y_* versus the constant *A* for various values of ξ for a water droplet placed on a vertical solid surface and γ = 0.072 *N*/*m*. The values *θ*_0_ = 60°, *P_L_* = 144 Pa, *r* = 2 mm, *d*_1_ = 1 mm and *d*_2_ = 1 mm were taken.

The balance equations for ice particle on a tilted surface are (Supplementary; Ice force and moment balance) 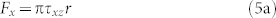

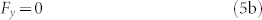

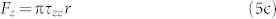

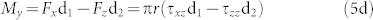
where *τ_xz_* is the shear stresses between ice and the solid surface applied in the *x*-direction. *τ_zz_* is the distributed normal stress which balances the torque created by the shear force *F_x_* applied at the offset d_1_.

When the adhesion force of ice to the solid surface is strong, fracture may occur within the ice itself; otherwise fracture can occur at the solid-ice interface where the cracks are usually presented^24^. Depending on whether normal or shear loading is predominant, the fracture scenario is different so that the former causes mode I (crack opening) and the latter makes mode II (edge sliding)^25^. The fracture in mode I usually occurs when the distributed normal stress overcomes the critical strength which is given by 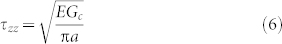
where *E* is the Young's modulus of the ice, *G_c_* is the surface energy of the crack and *a* is the crack length. The analysis for mode II crack fracture is similar. The value of surface energy depends on whether two surfaces are approaching or separating. The energy needed to separate surfaces is greater than that gained by bringing them together and the difference is the so-called adhesion hysteresis^26^. The adhesion hysteresis is one of the causes for CA hysteresis^6^,^27^.

According to Young's equation, the surface energy, *G_c_*, is given by 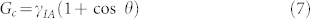
where 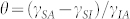 and *γ_SA_*, *γ_SI_* and *γ_IA_* are the solid-air, solid-ice and ice-air interfacial energies. During the opening of the crack (mode I), only the energy of separation which is related to the receding CA matters and therefore the surface energy is given by 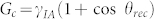. The energy needed to bring the surfaces together is related to the advancing CA. Substituting the value of surface energy into equations (6–7) yields 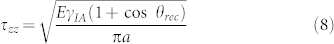
From this equation we conclude that the effect of crack size, *a*, on critical strength is especially significant when *θ_rec_* → 180°, which corresponds to superhydrophobic surfaces. For hydrophilic rough surfaces, since water penetrates to the cavities between asperities, they are usually in Wenzel state and therefore their adhesion to ice is stronger comparing to hydrophobic surfaces which are mostly in the Cassie state, with air pockets trapped between the solid and water droplet. When water is frozen, the cavities become air voids and can serve as stress concentrators.

**Figure 3:**
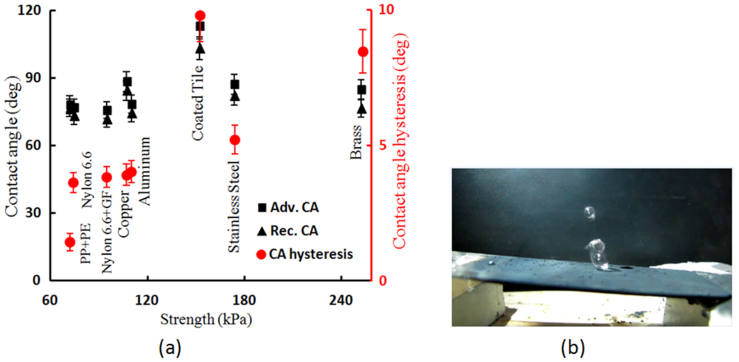
Icephobicity and hydrophobicity. (a) Advancing, receding CA (black) and CA hysteresis (red) of water on various samples vs. the strength of ice adhesion (b) impacting droplet test on a tilted soot-coated hydrophobic glass sample.

We experimentally studied the adhesion strength of ice on four metallic samples of aluminum, copper, brass 84400, stainless steel, and four non-metallic samples of nylon 6.6, nylon 6.6 + glass fiber, co-polypropylene (PP + PE) and TiO_2_ coated tile and also measured CA hysteresis of water droplet on the same samples as well as the surface roughness (with Phase II Surface Roughness Tester SRG-4500) for all samples except the tile, which was above the resolution (Table 1). The experiments were repeated 3 times for each sample and the error bars show the variation of these data. The results showed that the highest CA corresponds to the tile in non-metallic samples which also has the highest shear strength of ice indicating that the superhydrophobic or hydrophobic materials are not necessarily icephobic. However, the opposite behavior is observed in metallic samples. The highest CA corresponds to the copper which has the lowest shear strength of ice (Fig. 3a). The lowest CA hysteresis is on the PP + PE in non-metallic samples and copper in metallic samples which both have the lowest adhesion strength to the ice. As far as the correlation of the shear strength with surface roughness, it is observed from Table 1 that smoother samples tend to correspond to higher strength, which can be attributed to lower probability of interface void and crack formation in these samples.

The results show that CA hysteresis correlates with the ice adhesion strength on the hydrophilic samples (Fig. 3b). However, for the adhesion strength did not correlate with the receding contact angle, as it was described in the literature earlier^1^,^2^,^6^, but rather low adhesion strength correlated with low CA hysteresis.

Besides the parallelism between the dewetting and deicing, the effect of impacting water droplets on superhydrophobic surfaces below the water freezing temperature was investigated. A superhydrophobic surface was produced by coating glass substrate by soot. The water CA with the soot coated glass was 127°. The surface was kept for 5 minutes in the freezing room at −22°C. A syringe filled with tap water at 3 ± 2°C was used to drop the water on the substrate from the height of about 5 cm. The volume of each droplet was about 7 μl. It was observed that the droplets did not stick to the substrate, but bounced off the surface (Fig. 3b) Thus the ability of a superhydrophobic surface to bounce off incoming droplets reduces the time of contact with the solid, so that there is not enough time for water droplet to freeze (Supplementary; Water droplet impacting test).

The results demonstrated some correlation between the hydrophobic and icephobic properties as defined in terms of local adhesion forces and the effect of incoming water droplet.

## Discussion

Our results are consistent with the concept of the parallelism between the definitions of the superhydrophobicity and icephobicity (Table 2). Thus, the requirement of low surface energy and high CA for superhydrophobic surfaces corresponds to low solid-ice adhesion and low normal strength in the icephobicity. Low CA hysteresis corresponds to shear loading and thus to low shear strength in icephobic surfaces. The condition of bouncing-off incoming droplet is similar in superhydrophobicity and icephobicty. Finally, delayed crystallization of ice corresponds to the “antifogging” property of superhydrophobic surfaces.

All these features should be considered for a universal and robust definition of the icephobicity, which has been a matter of recent discussion in the literature^28^. We therefore, suggest that a surface should be called icephobic if it delays ice formation from condensed or incoming water in the situation where normally ice would form (i.e., at the temperatures below water freezing point) and/or if it has weak shear and normal adhesion strength to ice (the threshold of 100 kPa can be suggested, although the value is somewhat arbitrary, similarly to how the superhydrophobicity is defined for CA > 150°).

**Figure 4:**
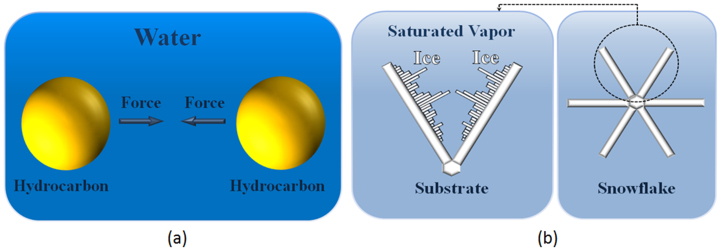
Parallelism of hydrophobic interaction and ice formation. (a) The attractive force between hydrophobic molecules (hydrocarbons) is a result of Gibbs energy ΔG = ΔH − TΔS minimization due to entropy increase ΔS in water molecular network (hydrocarbon-water-hydrocarbon system) prevailing over enthalpy ΔH. (b) The synchronization of branch growth during formation of a snow crystal due to ΔS prevailing over ΔH (ice-saturated vapor-ice system).

Besides wetting, hydrophobicity is crucial for many important effects, such as the “hydrophobic effect” and hydrophobic interactions. For two hydrophobic molecules (e.g., hydrocarbons) placed in water, there is an effective repulsive hydrophobic force due to their interaction with the water medium. The hydrophobic effect is responsible for folding of proteins and other macro-molecules (Fig. 4a).

To further stress the similarity between the hydrophobicity and icephobicity, it is noted that a special type of self-organized behavior, so-called self-organized criticality (SOC), may result from hydrophobic folding of proteins which is evidenced by power law exponents of the accessible surface area correlated with the hydrophobicity scales in protein amino-acids^29^,^30^. SOC is the major mechanisms with creates complexity in many systems and it has a specific “signature” on the quantitative characteristics of the system, such as the “one-over-frequency” noise, power exponents and fractalness^31^. Protein folding is a typical example of hydrophobically self-organized criticality^29^,^30^.

Are there any effects with similar signature related to the icephobicity? An obvious candidate is snowflake crystallization. Snowflakes are known to have fractal shape. Furthermore, their shape is very diverse with “no two flakes similar to each other.” On the other hand, many snow crystals are symmetric with each of the six branches almost identical to other five branches (Fig. 4b). The perceived “synchronization” of branch growth is stipulated by the history of the flake formation. The snow crystals grow in oversaturated vapor under negative temperatures, whereas the direction of their crystal growth is very sensitive to the variation of the temperature *T* and humidity (or oversaturation density *ρ*) varying from prisms to thin, solid, and sectored plates to dendrites to needles and columns. Each of these shapes is characterized by a fractal dimension *D*(*T, ρ*). When a snowflake falls, it passes through a unique history of temperature *T*(*t*) and humidity *ρ*(*t*) which defines the unique shape of a branch, however, similar for all six branches of the crystal. While perhaps one can speak about the “icephobic interaction” only in a metaphoric sense, the essence of the effect – the apparent “synchronization” of remote branches due to the interaction with the medium (oversaturated vapor) – is somewhat similar to the hydrophobic effect – the apparent repulsion of the hydrophobic particles due to their interaction with the medium (water). Furthermore, both effects can lead to quite complex phenomena, such as SOC-driven complexity as a result of hydrophobic interactions (during wetting of rough/heterogeneous surfaces or during polypeptide chain folding and looping^29^,^30^) or ice crystallization (fractal snowflakes)^32^.

Note that thermodynamically both the hydrophobic interactions and ice formation are driven by the minimization of the Gibbs surface energy, Δ*G* = Δ*H* − *T*Δ*S*. This is because in the hydrophobic interactions large positive value of *T*Δ*S* prevails over a small positive value of Δ*H* making spontaneous hydrophobic interaction energetically profitable^26^. The so-called surface roughening transition^33^ governs the direction of ice crystal growth and occurs at the critical temperature, above which the entropic contribution into the Gibbs energy, *T*Δ*S*, prevails over the enthalpic contribution, Δ*H*, thus making it more energetically profitable for the ice crystal to be rough rather than smooth^33^. This suggests that thermodynamically both the icephobic and hydrophobic behaviors can be viewed as entropic effects.

In conclusion, we investigated the parallelism between the hydrophobicity and icephobicity and suggested a definition of icephobicity which combines various requirements for anti-icing surfaces, namely, weak adhesion with the solid substrate and the ability to repel incoming droplets. We conducted a comparative study of wetting and anti-icing behavior. The theoretical force analysis shows that the main parameter affecting droplet adhesion to a solid surface is CA hysteresis, while for ice particles both receding CA and the size of voids/defects are important. However, in practice ice adhesion does always not correlate with the receding CA or with CA hysteresis. This parallelism between the hydrophobicty and icephobicity is in how these phenomena are defined: the key parameters are the normal and shear strength for icephobic surfaces and the CA and CA hysteresis for hydrophobic surfaces. Furthermore, the thermodynamic descriptions of the hydrophobic interaction and icephobicity may be similar. This is because both of them depend on the entropic term, −*T*Δ*S*, in the Gibbs free energy which dominates over the enthalpic term, Δ*H*, during the hydrophobic interaction and also governs the roughening transition during ice formation. The effects of the hydrophobic interactions and icephobicity are also similar since both can involve the self-assembly of new structures such as snow crystals or complex molecules (e.g., protein folding). Therefore, besides the practical application of the icephobicity to design novel ice-repellent surfaces, the phenomenon is also of interest from the theoretical point of view.

## Methods

The most common method to measure ice adhesion to the surface is applying a compressive or tensile force resulting in shear stress on the ice confined between two surfaces. Cylindrical or rectangular ice samples can be used. In order to measure the adhesion force of ice to different materials, we used a PASCO CI-6746 stress/strain apparatus 750 interface equipped with an economy force sensor (see supplementary information figure). The DataStudio software was used to record and analyze the data. We used various samples as the substrates and let the water to freeze on the samples using a plastic cylindrical mold. The sample was placed in the apparatus and the horizontal shear force was applied to the base of the ice column through a ring set around the ice and by rotating the apparatus handle until the ice was separated from the substrate (see supplementary information figure).

We prepared eight samples. The metallic samples were polished with a soft cloth impregnated with 1 μm silica and then were washed and cleaned with deionized water and finally were air dried. In order to freeze water column on substrates surface, thin plastic tubes with 5 mm inside diameter and 10 mm height were used as molds. The plastic molds were placed vertically on substrates surface and a Permatex black silicone sealant was gently applied to the outside surface of mold's base on substrates to prevent water leakage.

The molds were then filled with water using a syringe and left inside the freezing room at −20°C until the water was entirely frozen, and then the sealant was removed.

In order to measure the force applied to the ice column, each sample was transferred separately to the other freezing room with the temperature of −3 ± 2°C where the stress/strain apparatus was located. Then the horizontal shear force was applied to the base of the ice column until it was separated from the substrate (see supplementary information figure).

The magnitude of the applied force was recorded by a computer located outside the freezing rooms using the DataStudio software. In order to investigate the wetting behavior of above-mentioned samples, the static advancing and receding water CAs were measured using a Ramé-Hart 250 goniometer/tensiometer. The water CA, CA hysteresis and the shear strength of ice on various samples are present in Table 1.

## Author Contributions

M.N. suggested the idea, supervised the research and prepared most of the manuscript text, V.H. prepared metallic samples, conducted the experiments, developed the model and prepared the figures, K.S. prepared the non-metallic samples and supervised freezing chamber experiments. All authors reviewed the manuscript.

## Supplementary Material

Supplementary Information Supplementary material

## Figures and Tables

**Table 1:** Water CA of the samples and shear strength of the ice

Material		Surface roughness (μm)	Max. Force (N)	Strength (kPa)	Water CA°	Adv. CA°	Rec. CA°	CA hysteresis°
Metallic	Aluminum	0.6	2.16	110	78.01	78.39	74.33	4.06
	Copper	0.076	2.1	106.95	85.26	88.34	84.41	3.93
	Brass	0.047	4.95	252.1	83.80	84.87	76.38	8.49
	Stainless Steel	0.32	3.41	173.67	83.57	87.22	81.99	5.23
Non-Metallic	TiO_2_ coated tile	>10	2.99	152.28	111.38	113.03	103.21	9.82
	Nylon6.6	0.058	1.46	74.36	74.34	76.79	73.14	3.65
	Nylon6.6 + GF	0.7	1.86	94.73	73.43	75.71	71.85	3.86
	PP + PE	1.19	1.41	71.81	77.85	78.10	76.66	1.44

**Table 2:** The comparison of the hydrophobicity and icephobicity

Property	Water	Ice
Definition of “phobicity”	Low surface energy/low adhesion	Low adhesion
	High contact angle	Low normal strength (maximum stress)
	Low CA hysteresis	Low shear strength
	Bouncing-off	Bouncing-off supercooled droplets
Interaction	Hydrophobic interaction	Crystallization of ice and snowflakes
Thermodynamic relationship	Minimization of ΔG = ΔH − TΔS	Surface roughening transition ΔG = ΔH − TΔS = 0 and similar
Typical manifestation of the interactions	Hydrocarbone molecules in water environment	Ice molecules in saturated vapor environment at negative temperatures
Effects	Protein folding (including fractal shape), self-organized criticality, long-range hydrophobic force, wetting transitions	Snowlakes (diverse but symmetric, fractal)
