# Supplementary material for: From superhydrophobicity to icephobicity: forces and interaction analysis
Source: Sci Rep. 2013 Jul 12;3:2194. doi: 10.1038/srep02194 (PMC3709168; doi:10.1038/srep02194)
Supplement: Supplementary Information — Supplementary material [file srep02194-s1.pdf]

From superhydrophobicity to icephobicity: force and interaction analysis

Vahid Hejazi, Konstantin Sobolev, and Michael Nosonovsky*

*Correspondence to nosonovs@uwm.edu
College of Engineering & Applied Science, University of Wisconsin-Milwaukee,
Milwaukee, Wisconsin 53211, United States

Supplementary Information

Surface tension

Surface tension of a droplet placed on a solid surface can be presented in the vector form

$$\vec{\gamma}(\alpha) = \gamma_x \vec{\imath} + \gamma_y \vec{\jmath} + \gamma_z \vec{k} \qquad \text{(a)}$$

where γ_x, γ_y and γ_z are x-, y- and z-component of water-air interfacial energy and given by

$$\gamma_x = \gamma cos\theta sin\alpha \qquad \text{(b)}$$

$$\gamma_y = -\gamma cos\theta cos\alpha \qquad \text{(c)}$$

$$\gamma_z = \gamma sin\theta \qquad \text{(d)}$$

Substituting equations (b), (c), and (d) in equation (a) yields equation (2).

CA hysteresis

The receding and advancing CAs on the tilted surface are given by substituting $\alpha = \pi/2$ and $\alpha = 3\pi/2$ into equation (1), respectively

$$\cos\theta_{rec} = \cos\theta_0 + A\sin\psi \qquad \text{(e)}$$

$$\cos\theta_{adv} = \cos\theta_0 + A(\xi - 1)\sin\psi \qquad \text{(f)}$$

Force and moment balance

Considering the free body diagram of the water droplet placed on the surface, the external forces F_x, F_y, and F_z applied in x, y, and z direction, the surface tension force of water droplet acting upon the triple line, and the reaction force of the surface to the Laplace pressure inside water droplet applied to the solid-liquid wetting area are present. One can assume that F_x, F_y, and F_z are the gravity forces applied to the center of gravity of the water droplet, G, as $F_x = Mgsin\psi$, $F_y = 0$ and $F_z = -Mgcos\psi$. The gravity force has no component in y direction. M is the mass of the droplet and g is the acceleration of gravity. The Laplace pressure has no components in x and y directions because it is applied perpendicular to the surface, therefore it should be considered only in z direction equation of force balance. Equations of balance of the forces and the moments per unit length can be presented as

$$F_x + \int_0^{2\pi} \gamma_x \, d\alpha = 0 \qquad \text{(g)}$$

$$F_y + \int_0^{2\pi} \gamma_y \, d\alpha = 0 \qquad \text{(h)}$$

$$F_z + \int_0^{2\pi} \gamma_z \, d\alpha + r \int_0^{2\pi} p(x) cos^2 \alpha \, d\alpha = 0 \qquad \text{(i)}$$

where $p(x) = (P_L + \rho g x)$ is the Laplace pressure applied to the solid-liquid interface. Laplace pressure is inversely proportional to the local droplet curvature. The difference in the local curvature of the droplet induced by tilting the surface, results in change in internal Laplace pressure at each point at the solid-liquid interface. Since the droplet is in an equilibrium configuration, the forces are balanced. Substituting equation (1) into equation (b) and the then into equation (g) yields

$$F_x = -\int_0^{2\pi} \gamma \left(\cos\theta_0 + \frac{A}{2} \sin\psi \left[\xi + (2-\xi)\sin\alpha\right]\right) sin\alpha \, d\alpha \qquad \text{(j)}$$

The y-components of surface tension at the triple line are balanced due to the symmetry, therefore $F_y = 0$.

Assuming $x = -rsin\alpha$ and $y = rcos\alpha$, substituting equation (d) into equation (i) and considering the value of $p(x)$ yields

$$F_z = -\int_0^{2\pi} \gamma sin\theta \;\, d\alpha - r \int_0^{2\pi} (P_L - \rho g r sin\alpha) cos^2 \alpha \, d\alpha \qquad \text{(k)}$$

Calculating the second integral, considering the value of $\sin\theta = (1 - \cos^2\theta)^{1/2}$ and substituting equation (1) into equation (k) gives

$$F_z = -\int_0^{2\pi} \gamma \sqrt{1 - \left(\cos\theta_0 + \frac{A}{2} \sin\psi \left[\xi + (2-\xi)\sin\alpha\right]\right)^2} \, d\alpha - \pi P_L r \qquad \text{(l)}$$

where r is the radius of the droplet and P_L is the average Laplace pressure applied to the solid-liquid interface of a horizontal surface.

Assuming $f(\alpha) = \sqrt{1 - \left(\cos\theta_0 + \frac{A}{2} \sin\psi \left[\xi + (2-\xi)\sin\alpha\right]\right)^2}$, The value of F_z can be obtained as

$$F_z = -\gamma \int_0^{2\pi} f(\alpha) \, d\alpha - \pi P_L r \qquad \text{(m)}$$

For small values of A, $f(\alpha)$ can be approximated as

$$f(\alpha) = \sin\theta_0 + \frac{\cos\theta_0 \sin\psi (\xi + (2-\xi)\sin\alpha)}{2\sin\theta_0} A \qquad \text{(n)}$$

Equations of balance of the moments per unit length can be presented as

$$M_y = F_x \mathrm{d}_1 - F_z \mathrm{d}_2 - r^2 \int_0^{2\pi} p(x) \sin\alpha \, cos^2 \alpha \, d\alpha - r \int_0^{2\pi} \gamma_z \sin\alpha \, d\alpha \qquad \text{(o)}$$

$$M_x = M_z = 0 \qquad \text{(p)}$$

where d_1 and d_2 are the distances from the point G to the x and z axes, respectively (Fig. 2c). Equation of moment (Equation 4d) can be obtained by substituting equations (4a) and (4c) into equation (o).

For the water droplet placed on the tilted surface, considering equations (4a), (4c) and (4d) and assuming $d_1 = 3r/8$, the balance of forces and the moment in matrix form can be given as

$$\begin{bmatrix} 0 & \frac{\pi}{2}\gamma & 0 & 0 & 0 \\ -\frac{2\pi\gamma\cos\theta_0}{\sin\theta_0}\sin\psi & -\frac{\pi\gamma\cos\theta_0}{\sin\theta_0} & 0 & 0 & -\pi P_L r - 2\pi r\sin\theta_0 \\ 0 & \frac{3\pi}{16}r\gamma & \pi P_L r + 2\pi r\sin\theta_0 & \frac{\pi\xi\cos\theta_0}{\sin\theta_0}\sin\psi & -\frac{\pi\rho g r^3}{4} \end{bmatrix} \times$$

$$\begin{bmatrix} A \\ A\,(\xi - 2)\sin\psi \\ \mathrm{d}_2 \\ A\mathrm{d}_2 \\ 1 \end{bmatrix} = \begin{bmatrix} F_X \\ F_z \\ M_y \end{bmatrix} \quad \text{(q)}$$

Ice force and moment balance

To write the balance equations of forces, one has to consider the shear force applied to the solid-ice interface. Considering the shear force applied to the solid-ice interface, the balance equations of force are given as

$$F_x + \beta \int_0^{2\pi} \gamma_x \, d\alpha - \delta\pi\tau_{xz} r = 0 \quad \text{(r)}$$

$$F_y + \beta \int_0^{2\pi} \gamma_y \, d\alpha + \delta\pi\tau_{yz} r = 0 \quad \text{(s)}$$

$$F_z + \beta \left(\int_0^{2\pi} \gamma_z \, d\alpha + r \int_0^{2\pi} p(x) cos^2\,\alpha \, d\alpha \right) + \delta\pi\tau_{zz} r = 0 \quad \text{(t)}$$

where τ_{yz} is the shear stresses between ice and the solid surface applied in y direction. The value of τ_{yz} is zero due to symmetry. β and δ are the coefficients which determine the phase of deposited object so that for water $\beta = 1$, $\delta = 0$ and for ice $\beta = 0$, $\delta = 1$. Equations (r), (s) and (t) can be applied to both water and ice deposited on the solid surface.

The balance equation of the moment of ice about the point O at the center of the solid-ice interface (Fig. 2d) is given by

$$M_y = F_x \mathrm{d}_1 - F_z \mathrm{d}_2 = \pi r(\tau_{xz}\mathrm{d}_1 - \tau_{zz}\mathrm{d}_2) \quad \text{(u)}$$

Equations of the balance of forces and the moment for ice placed on vertical surfaces in matrix form can be given as

$$\begin{bmatrix} \pi r & 0 & 0 \\ 0 & \pi r & 0 \\ 0 & 0 & \pi r \end{bmatrix} \times \begin{bmatrix} \tau_{xz} \\ \tau_{zz} \\ \tau_{xz}\mathrm{d}_1 - \tau_{zz}\mathrm{d}_2 \end{bmatrix} = \begin{bmatrix} F_x \\ F_z \\ M_y \end{bmatrix} \quad \text{(v)}$$

Methods

The most common method to measure ice adhesion to the surface is applying a compressive or tensile force resulting in shear stress on the ice confined between two surfaces. Cylindrical or rectangular ice samples can be used. In order to measure the adhesion force of ice to different materials, we used a PASCO stress/strain apparatus 750 interface equipped with an economy force sensor (PASCO CI-6746, Supplementary Fig. a). The DataStudio software was used to record and analyze the data. We used various samples as the substrates and let the water being frozen on them using a plastic cylindrical mold. The sample was placed in the apparatus

and the horizontal shear force was applied to the base of the ice column through a ring set around the ice and by rotating the apparatus handle with uniform velocity until the ice was separated from the substrate (Supplementary Fig. b). The dependency of the force vs. the time of deformation (approximately proportional to the displacement) was recorded by the computer (Supplementary Fig. c).

We prepared eight samples. The metallic samples were polished with a soft cloth impregnated with 1 μm silica and then were washed and cleaned with deionized water and finally were air dried. In order to freeze water column on substrates surface, thin tubes of plastic cut from a common drinking straw with 5 mm inside diameter and 10 mm height were used as molds. The plastic molds were placed vertically on substrates surface and a Permatex black silicone sealant was gently applied to the outside surface of mold’s base on substrates to prevent water leakage. The surface roughness was measured with Phase II Surface Roughness Tester SRG-4500)

The molds were then filled with water using a syringe and left inside the freezing room at -20 ^{o}C until the water was entirely frozen, and then the sealant was removed.

In order to measure the force applied to the ice column, each sample was transferred separately to the other freezing room with the temperature of -5 to -1 ^{o}C where the stress/strain apparatus was located. Then the horizontal shear force was applied to the base of the ice column until it was separated from the substrate (Supplementary Fig. b).

The magnitude of the applied force was recorded by a computer located outside the freezing rooms using the DataStudio software.

In order to investigate the wetting behavior of above-mentioned samples, the static advancing and receding water CAs were measured using a ramé-hart 250 goniometer/tensiometer (Supplementary table).

Supplementary table Water CAs of the samples and shear strength of the ice

Material		Max. Force (N)	Strength (kPa)	Water CA ^{o}	Adv. CA^{o}	Rec. CA^{o}	CA hysteresis^{o}
**Metallic**	Aluminum	2.16	110	78.01	78.39	74.33	4.06
	Copper	2.1	106.95	85.26	88.34	84.41	3.93
	Brass	4.95	252.1	83.80	84.87	76.38	8.49
	S.S.	3.41	173.67	83.57	87.22	81.99	5.23
**Non-Metallic**	Tile	2.99	152.28	111.38	113.03	103.21	9.82
	Nylon6.6	1.46	74.36	74.34	76.79	73.14	3.65
	Nylon6.6+GF	1.86	94.73	73.43	75.71	71.85	3.86
	PP+PE	1.41	71.81	77.85	78.10	76.66	1.44

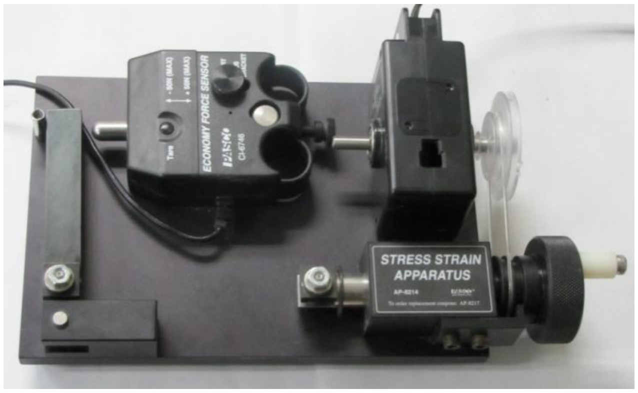

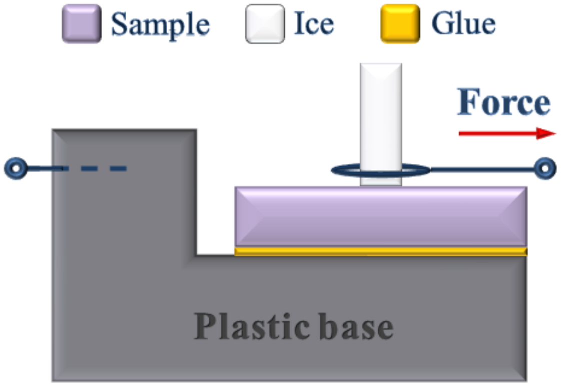

(a) (b)

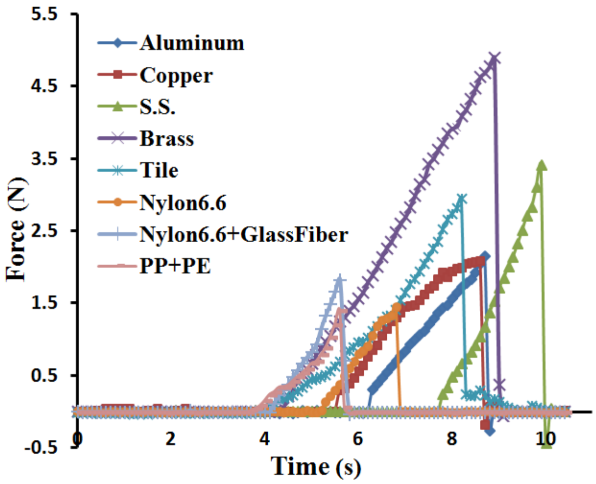

(c)

Supplementary figure 1. Schematic of the apparatus (a) PASCO stress/strain apparatus (b) Horizontal force applied to the ice column (c) Force versus the time of deformation / distance as recorded by the computer (these dependences essentially constitute stress-strain curves). Color lines show the applied forces to the ice on various substrates versus time

Water droplet impact tests

A hydrophobic surface was produced by coating glass with soot. The water CA with the sample was 127°. The sample was kept for 5 minutes in the freezing room at -22 °C. A syringe filled with the tap water at 3 °C was used to drop the water on the substrate from the height of 5 cm. The volume of each droplet was about 7 µl. It was observed that the droplet did not stick to the substrate and bounced off the surface.

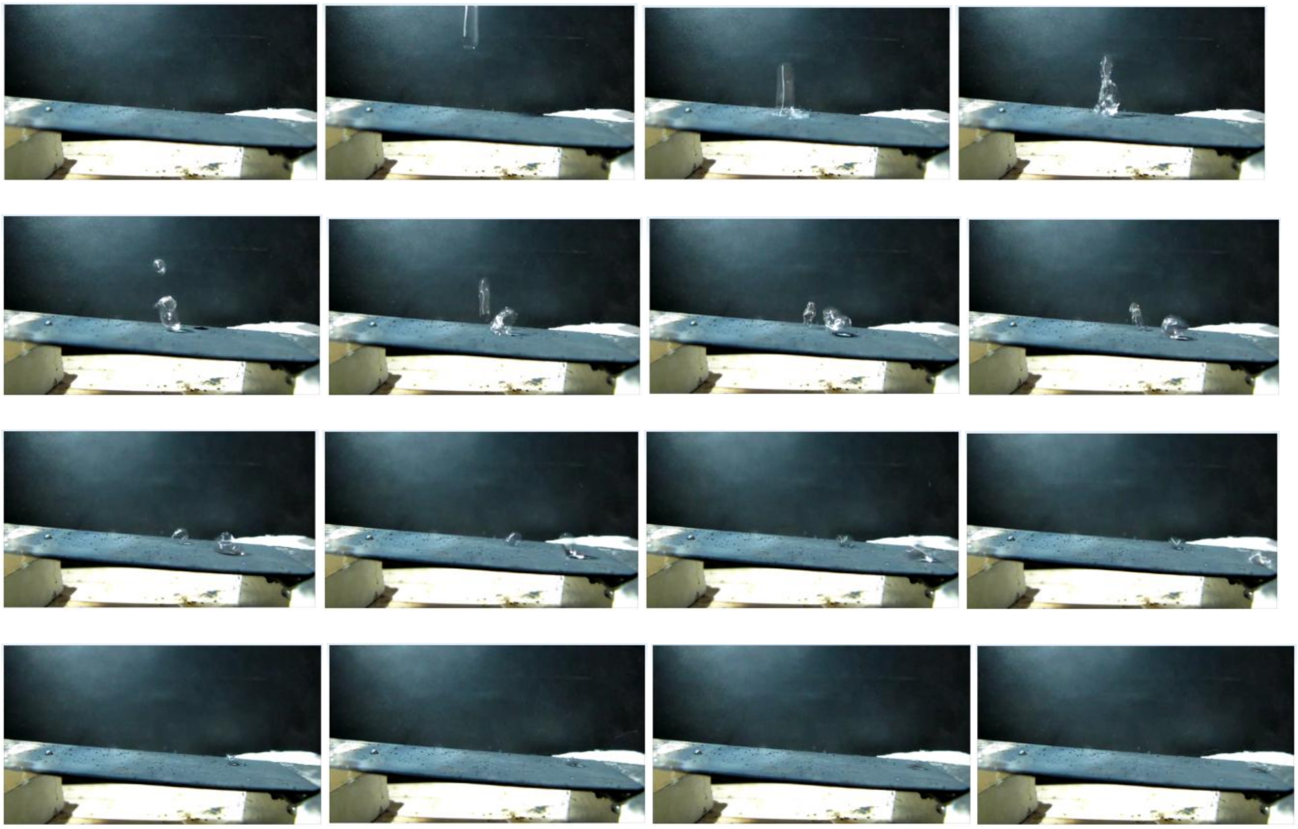

Supplementary figure 2. A tilted glass substrate coated by soot was kept for 5 minutes in freezing room at -22°C. A syringe filled with the 0-5°C tap water was used to drop the water on the substrate. The height of the distance between the needle tip and the substrate was about 5 cm and the volume of each droplet was about 7 µl. The static contact angle of the water droplet on the soot coated glass was 127°. It was observed that the droplet does not stick to the substrate and jumps off the surface.

A tilted glass substrate coated by soot was kept for 5 minutes in freezing room at -22°C. A syringe filled with the 0-5°C tap water was used to drop the water on the substrate. The height of the distance between the needle tip and the substrate was about 5 cm and the volume of each droplet was about 7 µl. The static contact angle of the water droplet on the soot coated glass was 127°. It was observed that the droplet does not stick to the substrate and jumps off the surface.
